# Manganese, a Likely Cause of 'Parkinson's in Cirrhosis', a Unique Clinical Entity of Acquired Hepatocerebral Degeneration

**DOI:** 10.7759/cureus.10448

**Published:** 2020-09-14

**Authors:** Zainab Mehkari, Lubna Mohammed, Moiz Javed, Aldanah Althwanay, Farah Ahsan, Federico Oliveri, Harshit K Goud, Ian H Rutkofsky

**Affiliations:** 1 Internal Medicine, California Institute of Behavioral Neuroscience & Psychology, Fairfield, USA; 2 Internal Medicine, California Institute of Behavioral Neurosciences & Psychology, Fairfield, USA; 3 Cardiology, California Institute of Behavioral Neurosciences & Psychology, Fairfield, USA; 4 Psychiatry, Neuroscience, California Institute of Behavioral Neurosciences & Psychology, Fairfield, USA

**Keywords:** parkinsons disease, liver cirrhosis, neurological manifestation of chronic liver disease, manganese and dopamine, parkinson cirrhosis, manganese and chronic liver disease, acquired hepatocerebral degeneration, acquired hepatolenticular degeneration, manganese and dopamine transporter, dopamine in liver cirrhosis

## Abstract

With idiopathic Parkinson's disease being a common entity, parkinsonism in acquired hepatocerebral degeneration (AHD) in the context of Manganese (Mn) has gained importance in recent years. An insight into the pathomechanisms behind this disease has been put forth. How can Mn as a divalent metal exert its effect in leading to chronic neurodegenerative disorder? Secondary to decreased excretion in liver cirrhosis, Mn significantly alters the striatal dopaminergic system. Management of this debilitating disease also focuses on different aspects where Mn has been involved in the pathogenesis. We have put forth the details behind Mn effects in Parkinson’s, which will be a guide for better understanding and management of this disease.

A literature search was performed using PubMed as a sole database, and all the articles were peer-reviewed. The author tried to follow the PRISMA guidelines. Inclusion criteria were set for 10 years, with most studies with in the last seven years. All types of study designs were included relevant to the topic, clearly delineating the pathomechanisms of Mn in the disease and also its management. After extensive research, through the PubMed database, we found that Parkinson's disease is one of the neurological complications in advanced liver cirrhosis. Mn is an essential element behind its pathogenesis; it works at different cellular levels to promote neurotoxicity. From its influx to its effects on dopamine transporters (DAT), where it disrupts dopamine homeostasis also altering postsynaptic dopamine (D2) receptors, it disrupts mitochondria and the endoplasmic reticulum (ER) promotes oxidative stress and neuroinflammation. Misfolding of alpha-synuclein (α-Syn) is promoted on chronic exposure to Mn where α-Syn from being neuroprotective becomes neurotoxic. It also alters glutaminergic and gabaergic neurotransmission. In a nutshell, the diversity of its effect on nigrostriatal denervation is challenging. The importance of neuroimaging and various approaches to management is also discussed. AHD, an uncommon entity in advanced liver cirrhosis, needs more awareness so that it can be diagnosed earlier and better therapeutic options can be sought. Our study highlighted Mn mechanisms behind this clinical entity, putting forth grounds for a better understanding of this disease. Advanced research targeting Mn for managing this disease will be revolutionary.

## Introduction and background

Acquired hepatocerebral degeneration (AHD) is a rare form of progressive and irreversible neurological disease occurring in advanced liver disease affecting predominantly the basal ganglia resulting in extrapyramidal (hypokinesia, dystonia, rigidity, dysarthria, intention tremor) and neuropsychiatric symptoms [1]. "Parkinson in cirrhosis” is also called AHD because of the specific involvement of the lentiform nucleus (globus pallidus and putamen), though there have been degenerative changes in other areas of basal ganglia also [2]. Symptom onset coincides with the duration of liver damage and occurs gradually over some time [3]. Because it gets easily confused with hepatic encephalopathy, it should always be on the list of differentials for neurological complications associated with chronic liver disease [4].

Prevalence

The prevalence of the disease is 21% [2]. A follow-up study was conducted to assess the development of parkinsonism in patients with advanced liver disease, the results of which were statistically significant showing a 2.65% increase in parkinsonism in cirrhotic patients [5]. A case report of a woman with cirrhosis presented with progressive neurological deterioration and worsening of extrapyramidal symptoms for three years, not responding to ammonia lowering therapy leads to the diagnosis of AHD when her symptoms deteriorated and magnetic resonance imaging (MRI) findings showed bilateral hyperintensity of the basal ganglia onT1-weighted MRI, which is a characteristic finding in Parkinson's cirrhosis [6]. Cirrhosis-related parkinsonism is more prevalent than thought [7].

Pathophysiology: an insight to the mechanism

The liver plays an important role in maintaining and clearing toxic chemicals from the body. Damage to the liver occurs due to several causes, the most common of which are viruses, alcohol, nonalcoholic steatohepatitis (NASH), autoimmune hepatitis, and biliary diseases. Failure of the liver to detoxify blood results in neurotoxins which include manganese (Mn), ammonia, lactate entering the cerebral circulation, this is further aggravated by the presence of portosystemic shunts commonly present in advanced liver disease or after transjugular intrahepatic portosystemic shunt (TIPS) [8]. A variety of neurologic complications occur in liver cirrhosis the cause of which is related to multiple factors like increased ammonia, Mn, and disrupted blood-brain barrier (BBB). A few neurologic diseases are hepatic encephalopathy, hepatic myelopathy, encephalitis, and demyelination, with cirrhosis-related parkinsonism being one of them [9]. Mn accumulation in the basal ganglia is the key factor involved in pathogenesis. At the synaptic level, it damages presynaptic dopamine transporters and postsynaptic dopamine (D2) receptors leading to the clinical features of ataxia and movement disorder [2]. Clinical features can be very diverse including extrapyramidal and cerebellar signs [10]. 

This article will discuss how advanced liver disease causes this rare debilitating disease with Mn deposition in the globus pallidus and striatum after it damages dopamine transporter (DAT), which plays an important role in maintaining dopamine levels via dopamine efflux at the synaptic level [11,12], and how it acts at the cellular level disrupting mitochondrial function and generating reactive oxygen species (ROS) causing widespread neuroinflammation and cell death [13]. T1 hyperintensity on MRI is necessary for clinical features of AHD signifying Mn deposition but is lacking complete evidence [1,14]. T1 hyperintensity usually occurs at internal pallidum but may be seen in striatum, cerebellum, and mesencephalon [8]. Thus making this entity clinically similar but pathologically different from idiopathic Parkinson’s disease where primarily dopaminergic neurons in the substantia nigra pars compacta are affected [15]. This also signifies the importance of performing MRI in patients with advanced chronic liver disease presenting with Parkinson’s symptoms [16,17]. Blood Mn levels are monitored serially but are not directly correlated with the symptom severity [14]. No particular treatment is available but studies have shown effective results with branched-chain amino acids, trientine, levodopa, bromocriptine, and liver transplant [8,2]. Liver transplant has shown promising results in eliminating extrapyramidal symptoms of this disease with the absence of palladium hyperintensity seen at six months of follow up [10,14,17]. Nonetheless, more studies need to be done to prevent and treat this debilitating illness.

## Review

Method

Search Strategy Protocol and Database Source

The author in this review article followed the Preferred Reporting Items for Systematic Review and Meta-Analysis (PRISMA) guidelines. PubMed was used as the sole database. The data was last collected on June 27, 2020. A comprehensive search was done related to the topic in finding how liver cirrhosis could lead to parkinsonism and how Mn plays a role and its intervention using the following keywords: 1) liver cirrhosis yielded 44,092, 2) Parkinson’s disease yielded 58,852, and 3) Neurological manifestation of chronic liver disease yielded 667 articles. Combining the keywords narrowed the search results to 4) 205 articles listed for manganese and dopamine, 5) 197 articles listed for Parkinson’s cirrhosis, 6) 69 articles listed for manganese and chronic liver disease, 7) 65 articles listed for acquired hepatocerebral degeneration, and 8) 48 articles listed for acquired hepatolenticular degeneration, finally yielding 9) 35 articles for dopamine in liver cirrhosis, 10) 21 articles for manganese and Parkinson’s cirrhosis.

Inclusion/Exclusion Criteria

All the articles selected were peer-reviewed and the author used robust inclusion criteria. Only studies done within the last 10 years were included, and most were done in the last seven years. The studies included were in the English language and no geographical restriction was applied. This article includes studies done in humans, animals (birds, oysters, rats), cadavers, cultured cells, and dopaminergic cell models to study the mechanism of Mn at the cellular level and its implication in the disease progression. There was no limitation on age, gender, or ethnicity. Data extracted included all types of study design, including a clinical trial, observational studies, case reports, systematic review, and meta-analysis. Relevant data from both the abstract and free full text was included. Grey literature, non-peer-reviewed, and duplicate studies were extracted. 

Research Strategy

**Figure 1:**
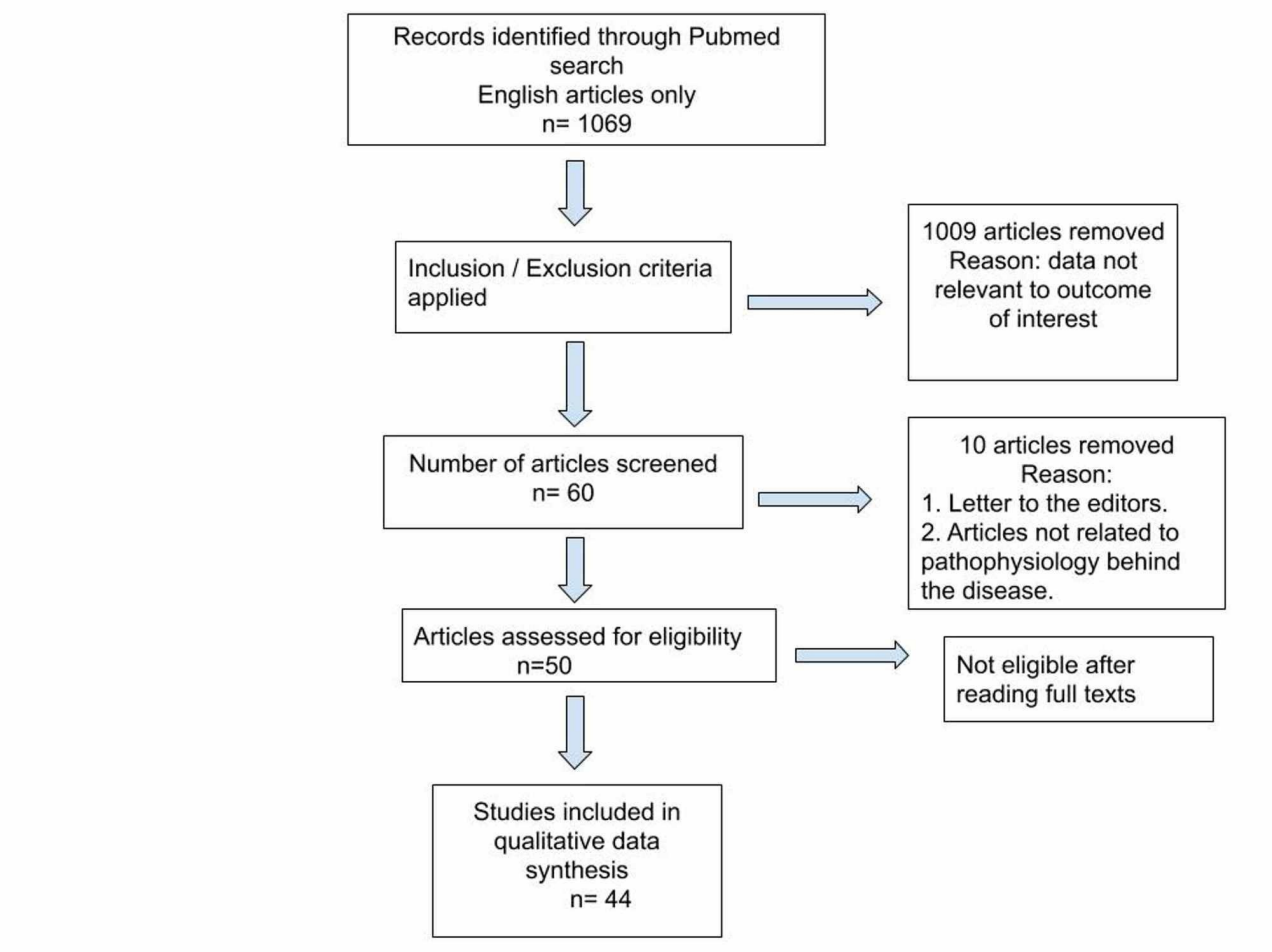
PRISMA flow diagram for study selection. PRISMA: Preferred Reporting Items for Systematic Review and Meta-Analysis

Out of extensive data search, we focused on the articles relevant to the topic which included neurological manifestation in advanced liver disease, how Mn plays a role in the pathogenesis, the diagnostic workup, and intervention (drugs and liver transplant). Clinical trials were included signifying the importance of liver transplant in the recovery of patients. Data about both the positive and negative outcomes were included. All the articles not relevant to the topic were excluded which included letters to the editors. The data was collected ethically and legally. A PRISMA flow diagram is depicted in Figure 1.

Results

We used Pubmed as our only database with the following results. Table 1 lists the number of articles and their corresponding keywords.

**Table 1:** Table of keywords. MeSH: Medical Subject Heading

Keywords	Database	Number Of Results
Parkinsons disease	Pubmed	58,852
Liver cirrhosis	Pubmed	44,092
Neurological manifestation of chronic liver disease	Pubmed	667
Manganese and dopamine	Pubmed	225
Parkinson cirrhosis	Pubmed	197
Manganese and chronic liver disease	Pubmed	69
Acquired Hepatocerebral degeneration	Pubmed	65
Acquired Hepatolenticular degeneration	Pubmed	48
Manganese and Dopamine transporter	Pubmed	41
Dopamine in liver cirrhosis	Pubmed	35
Manganese and Parkinson cirrhosis	Pubmed	21
Parkinson Disease [Mesh] and liver cirrhosis/complication [Mesh]	Pubmed	6
Liver cirrhosis [Mesh] and Parkinsonian disorder/pathophysiology [Mesh]	Pubmed	5

Out of 104,303, on PubMed, only the articles associated with Parkinson’s disease in advanced liver cirrhosis were studied, which were 1359, out of those only articles pertaining to the pathophysiology behind the disease and the intervention were selected. We excluded all other articles which were signifying other neurological diseases associated with liver cirrhosis and the duplicate studies, which narrowed down our search to 44 articles, out of which 13 were free full-text articles and 31 were abstracts; we included the studies referenced in those free full-text articles, which gave an in-depth study of pathomechanisms of Mn behind the disease. All the articles were peer-reviewed. Articles with all study designs were included, e.g. case reports, observational studies, clinical trials. We used PubMed as our sole data source but most of the articles were cross-referenced in Google Scholar also.

Discussion

**Figure 2:**
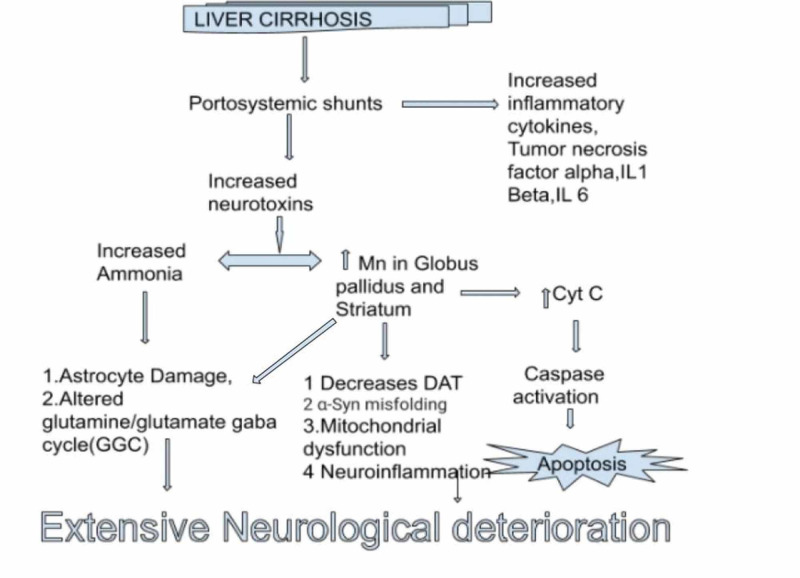
Role of manganese in acquired hepatocerebral degeneration. The idea for the figure was adopted from the article ' Recent Updates on Acquired Hepatocerebral Degeneration'. However, this figure was modified according to our study's requirement. Mn: manganese; Cyt-c: cytochrome C; DAT: dopamine transporter; IL1 beta: interleukin 1 beta; IL6: interleukin 6; α-Syn: alpha-synuclein

This mechanism by which advanced liver cirrhosis leads to Parkinson’s disease is complex since multiple etiologies combine to give this picture of a debilitating illness which is mostly irreversible. Accumulation of toxic substances ammonia, Mn along with oxidative stress, and widespread neuroinflammation predispose to the development of parkinsonism as shown in Figure 2 [3]. Clinical features of this illness coincide with characteristic Parkinson’s disease characterized by rigidity, dyskinesia, tremors, and neuropsychiatric manifestations since broadly the basal ganglia are involved with subtle changes [3,18]. Mn deposits preferably in globus pallidus and substantia nigra leading to hyperactivity of corticostriatal neurons [18].There are robust mechanisms involved in maintaining Mn levels in the body. It lies between 5.32 and 14.03 ng Mn/mg protein (20.0-52.8 μM Mn), whereas 15.96-42.09 ng Mn/mg protein (60.1-158.4 μM Mn) is the estimated pathophysiological threshold [19].

Furthermore, review studies by Shin et al. take into consideration how the liver in advanced liver disease is unable to detoxify toxic chemicals in the body leading to damage to the nigrostriatal pathway without causing degeneration of the dopaminergic neurons with clear deposition in globus pallidus and substantial nigra [3]. This as well as a case report conducted by Miletic et al. demonstrated how patients presented with gait disturbances and rigidity in advanced liver cirrhosis whose MRI demonstrated bilateral T1 hyperintensity [18], making this study more powerful since the actual patient-reported finding was observed. In vitro studies done by Bowman et al. gave an insight of Mn levels producing toxic effects [19]. As shown the margin for toxicity is very small, small changes in Mn homeostasis can lead to toxic levels, causing disruption of dopaminergic neurons.

1. *Mn and Its Pathogenesis at the Cellular Level*

The hepatobiliary system plays a major role in removing toxic substances from blood and cerebrospinal fluid (CSF), portosystemic shunting, and liver failure predispose them to enter the circulation. Liver failure is directly related to the toxic accumulation of Mn.

Regulation of Mn is intricately regulated with specific importers and exporters that maintain homeostatic Mn levels in the body. It can cross the BBB and also disrupts various neurotransmitters like gamma-aminobutyric acid (GABA), glutamate, and acetylcholine (Ach) in the synaptic cleft, leading to excitotoxicity seen in these patients. There are multiple mechanisms by which Mn induces toxicity.

2. *Altered Mn Influx Promoting Neurotoxicity*

Normally, in the presence of excess Mn, homeostatic mechanisms downregulate the uptake of Mn, but in chronic exposure, homeostatic mechanisms become deranged and a cascade of events occurs leading to neurodegeneration and neuroinflammation, which will be discussed.

High levels of Mn in the systemic circulation then enter the nervous system through these transporters. A literature review by Chen et al. showed that there are seven transporters that have an affinity to Mn. They are divalent metal transporter 1 (DMT1), DAT, transferrin receptor (Tfr), Zn transporters (Zip 8 and Zip 14), citrate transporters, choline transporters, and Ca channels (Ca+2) [20]. As the name suggests these transporters are involved in the transport of other metals. DMT1 and Tfr are of particular interest here. Mn mostly gets transported by DMT1. It can also diffuse through the cell membrane [21]. The role of these transporters is important in the context of Mn induced neurotoxicity.

2a. *Divalent Metal Transporter 1 (DMT1) and Transferrin Receptor (Tfr)*

DMT1 on the cell membrane opens up and transports Mn in its divalent form whereas there is endocytic uptake of the trivalent form (Mn+3) by Tfr [20,22]. The review articles by Chen et al. have described the morphology of these transporters while Gunter and his colleagues identified Tfr as the main transporter for Mn+3 in a study done on mice hippocampal and striatal neurons [20,22]. They used the fluorescent label on Mn+3 and Tfr complex which showed uptake of Mn+3 in an endosomal fashion close to the mitochondria.

Since patients with advanced liver disease are malnourished, iron deficiency anemia is a common lab finding, predisposing them to toxic effects of Mn by increased uptake via Tfr [20,23]. The observational study conducted by Smith EA et al on children in England showed a close relationship of how iron-deficiency anemia was linked with high Mn levels when they measured blood Mn levels, it was found to in the range of 11.7-42.4 μg/L, which are the toxic levels leading to neurological deterioration seen in those children. Making it a reliable study [23]. The study by Gunter et al was done on mice depicting the morphology of Tf receptors in Mn+3 uptake [22].

2b. *Effect of Mn at the Synaptic Junction: Dopamine Transporters (DAT)*

DAT are membrane-spanning proteins which play a crucial role in maintaining dopamine levels in the synaptic cleft, it is involved in the efflux of dopamine at the synaptic junction via the voltage-independent mechanism [11]. Mn disrupts dopamine homeostases at the presynaptic and postsynaptic levels without causing the degeneration of striatal neurons. It disrupts dopamine uptake via DAT and also disrupts amphetamine-induced dopamine efflux causing internalization of DAT eventually causing neurotoxicity [12,24]. The review articles by Kwakye et al. discussed the minute details of how DAT was effected by high Mn levels disturbing the dopamine homeostasis at the synaptic level [24], while Roth et al. researched human embryonic kidney cells (HEK cells) to show how Mn was toxic to the dopaminergic neurons along with dopamine giving an additive insult owing to its high intracellular concentration as a result of Mn exposure [12]. Since dopamine is chemically similar to catecholamines, it also has a propensity for oxidation yielding quinones and free radicals further exacerbating oxidative stress. Mn promotes dopamine’s autooxidation potential producing hydrogen peroxide (H_2_O_2_) [25].

Mn shares common transporters with other metals, taking into consideration how anemia causes upregulation of transferrin (Tfr) receptors, leading to an increased influx of Mn. Similarly, this literature review and a recent study by Roth et al. on HEK cells, where they used DAT transfected and control cells showed how Mn effects DAT, and Mn was found to be equally toxic to both the cell lines [12]. The dopamine transporter is downregulated by internalization leading to decreased dopamine reuptake and loss of dopamine in the synaptic cleft since DAT is involved in the reverse transport of dopamine which is the sole mechanism in recycling dopamine. This study seems more powerful as it delineated the effect of Mn on DAT and subsequent damage to the nigrostriatal pathway by dopamine. A literature review identified Mn promoting dopamine autooxidation and the release of reactive oxygen species further causing neurotoxicity. Mn also disrupts dopamine action on postsynaptic D2 receptors, but it is not clear how this effect occurs [2].

Both of the studies were done in the last seven years but the study on HEK cells seems more powerful. All of these additive effects lead to clinical features of parkinsonism (bradykinesia, rigidity, and cognitive deficits). Another recent review by Kwakye et al. demonstrated Mn-induced parkinsonism does not cause loss of dopaminergic neurons but does alter different homeostatic mechanisms making it pathologically different from idiopathic Parkinson's disease [24]. Although Mn also disrupts postsynaptic D2 receptors, as evidenced in a literature review by Butterworth but more research needs to be conducted to identify Mn effects on postsynaptic D2 receptors since it was a review done by a single author, more advanced level research needs to be conducted [2].

3. *Manganese And Oxidative Stress*

Accumulation of Mn occurs in globus pallidus and striatum, owing to its high oxygen consumption and Mn being a meal with high redox potential, it is neuroprotective acting as a cofactor for superoxide dismutase-2 (SOD-2). However, its chronic exposure and accumulation at high levels lead to neuronal cell apoptosis.

Given its divalent form, Mn can also bind to Ca+2 channels and enter the mitochondria [21]. Impaired mitochondrial activity as a result of Mn, induces oxidative stress and reactive oxygen species (ROS) are generated which triggers an apoptotic pathway where neuronal cell death occurs via the release of cytochrome C, which activates caspase-9, the initiator of apoptosis which cleaves caspase-3. The cleaved caspase then cleaves protein kinase C delta (PKCδ) leading to nuclear condensation and fragmentation of deoxyribonucleic acid (DNA) [26]. It was also found that Mn inhibits tyrosine hydroxylase (TH) the rate-limiting enzyme in dopamine synthesis through its effect on PKCδ [27]. Another recent study by Fernandes et al. on human neuroblastoma cell line SH-SY5Y, treated with manganese chloride (MnCl2) in the range from 0 to 100 μM periodically, showed how consumption of oxygen increased up to 10 μM and then decreased with concentrations above 50 μM [28]. The results showed how there was no superoxide (O2-) production by mitochondrial superoxide indicator (Mito Sox), while there was increased H_2_O_2_ produced as evidenced by mitochondria peroxy yellow 1 (Mito Py1), a fluorescent probe showing H_2_O_2_ production [28]. In another study done by Wang et al. on rats, where they divided 64 rats into four groups, injected MnCl2 intraperitoneally for four weeks (with 7.5, 15, and 30 mg/kg), using normal saline as a control, the results not only showed accumulation of Mn in the striatum but also loss of endoplasmic reticulum (ER) integrity, and ER-mediated apoptosis, caspase-9 and caspase-3 activations with decreased B-cell lymphoma (Bcl-2) expression, leading to neuronal apoptosis [29].

All of these studies point to how Mn goes from being neuroprotective at low doses to toxic at high doses and damages mitochondria, ER, and induces apoptotic factors, leading to dopaminergic cell death. All of these studies were done in the last five years on rats. Wang et al. followed up for four weeks, with continuous Mn exposure and identified the mitochondrial damage of the dopaminergic neurons in rats [29]. A study on human neuroblastoma cell lines by Fernandes et al. in 2017 also showed promising results as they used neuroblastoma cell lines which clearly explained neurodegenerative processes [28]. 

4.* Alpha-Synuclein and Mn*

The synuclein family is composed of alpha, beta, gamma synclines, alpha, and beta are found in the presynaptic neurons, while gamma in the glia [30], we will be focusing on alpha-synuclein (α-Syn), a pathological hallmark of various neurodegenerative diseases. α-Syn exhibits two roles in its exposure to Mn. 

4a. *Protective Role of Alpha-Synuclein:*

Its effect is modulated in a timely fashion, owing to its metal-binding sites on C-terminus where Mn binds to aspartic acid (Asp 121), asparagine (Asn 122), and glutamic acid (Glu 123) residues serving as a metal scavenger [13,21]. A recent study by Harischandra et al. on N27 dopaminergic neuronal cells on rats with human wild type α-Syn showed its protective effects for 24 hrs when compared to vector control cells via downregulation of the apoptotic cascade, by inhibiting the release of cytochrome C and eventually protecting against neurodegeneration promptly [13]. It also decreased Mn induced activation of PKCδ, involved in apoptosis whereas no changes were seen in ROS generation concluding alpha-synuclein as a storage site for Mn protecting against neurotoxic insult. The same effect was studied by Yan et al, where they used mice with wild type α-Syn gene knockout (α-Syn-/-) and wild-type (α-Syn+/+) when exposed to varying concentrations of Mn, made apparent the protective role of α-Syn at high Mn concentration (200 μmol/kg) making it a hub for Mn [31]. Furthermore, the results of both of these studies made clear the neuroprotective role of α-Syn in the initial stages of Mn exposure because of its metal-binding capacity, However, continuous exposure to Mn results in oligomerization of α-Syn downregulating its neuroprotective effect [13]. Another study by Ducic et al. revealed similar results in which midbrain neurons of rat, expressing α-Syn treated with Mn showed increased intracellular levels of Mn, while decreased levels of other metals, however, there were no alterations in other Mn transport proteins like DMT1, ferroportin 1 (Fpn1), and voltage-gated calcium channels (VGCC) [32], thus signifying the importance of alpha-synuclein acting as an intracellular store for Mn. 

4b. *Alpha-Synuclein Causing Neurotoxicity:*

Other studies have pointed out how α-Syn is associated with neurotoxicity by many other mechanisms in the presence of Mn. Xu et al. conducted a series of research on rat organotypic brain slices treated with Mn [33]. Initial results revealed oxidative stress was involved in mediating α-Syn oligomerization. Mn caused an increase in α-Syn messenger ribonucleic acid (mRNA) and protein expression causing membrane-bound oligomerization. Increasing the Mn concentration, Mn (II) chloride tetrahydrate, from 0-400 μM lead to increased neuronal apoptosis and reduced levels of superoxide dismutase (SOD) activity [33].

Another such study found Caplain 1, a protease used as a substrate by α-Syn, to be involved in signaling aggregation of it. Caplain is involved in cleaving α-Syn that then aggregates and form oligomers. After 24 hours of exposure to 400 μM Mn in rat brain slices, the rate of apoptosis increased by 29.6% along with increased levels of Ca, lactate dehydrogenase (LDH), and activity of the Caplain [34]. Protein disulfide isomerase, which is responsible for the proper folding and maturation of the protein, Xu et al. found that high Mn levels lead to nitrosative stress, through the S nitrosylation of protein disulfide isomerase and activation of inducible nitric oxide synthase (iNOS), in rat brain slices leading to the misfolding of α-Syn and subsequently decreasing its affinity to the protein disulfide isomerase, levels of nitric oxide (NO), rate of apoptosis, iNOS activity were directly related to the increasing Mn concentration [35]. α-Syn also has a role in DAT activity, it is involved in the downregulation of DAT by clathrin-mediated endocytosis, lowering its cell surface levels, this was tested by Ksios et al. in cultured cells and in mouse brains mimicking Parkinson’s disease when transgenic A532 α-Syn was used, results of which showed decreased uptake of dopamine from the synaptosome decreased recycling, and loss of dopamine eventually [36].

All these studies point to the fact that the Mn effect on α-Syn is time and concentration-dependent, being neuroprotective and acting as a scavenger initially, but with chronic exposure, it leads to misfolding, aggregation, and neurotoxicity. All the work was done in rats in the last seven years, showing a clear distinction between neurotoxicity and neuroprotection following acute and chronic exposures to Mn. Xu et al. showed neurotoxicity via ROS generation and S nitrosylation of protein disulfide isomerase leading to misfolding in a concentration-dependent fashion [35]. The study done by Harishchandra et al. is more convincing in signifying neuroprotection [13], while studies done by Xu et al. helped in understanding the neurotoxic effects of α-Syn at toxic Mn concentrations by varied mechanisms, causing damage to the plasma membrane, mitochondrial and ER membrane [33-35]. Apart from Mn, the α-Syn effect on DAT, further decreases effective dopamine at the synaptic cleft, leading to clinical features of parkinsonism [36]. These two studies were different in the context of using mutant and wild type α-Syn.

4c*. Role of Alpha-Synuclein in Promoting Neuroinflammation*

Misfolding of α-Syn in the presence of Mn is further exacerbated by the transport of this oligomerized protein in a prion-like fashion. Cell-to-cell transfer of this protein occurs in the presence of Mn as evidenced in a study done by Harischandra et al. on dopamine cultured neuronal cells, which showed misfolded α-Syn was excreted via exosomes into the extracellular space then taken up through the caveolae in the glial cells, mounting an inflammatory response and furthermore adding to the neurotoxicity in dopaminergic neuronal cells [37]. The transmission of these misfolded proteins expedites the neurodegeneration, as shown in another study using the dopaminergic cell model of Parkinson’s disease MN9D, expressing wild type α-Syn, Mn exposure caused the release of exosomes, with increased expression of Rab 27a, a protein involved in the regulation of exosome release, consequently, this and other micro ribonucleic acid (miRNA) expression was observed on Western blot [38].

Both of these studies were done in the last two years on a cultured cell model using wild type α-Syn, noting the importance of disease propagation via exosomes; the first study showed how exosomes when endocytosed will lead to neuroinflammation. Mn effect exacerbates cell-to-cell trafficking, and this can be helpful as a marker for disease identification and also be used as a target for treatment by using exosomes as drug-delivering machines in the future.

5. *Manganese and Neuroinflammation*

Neuroinflammation induced by Mn is still not clear, and varied mechanisms come into play. The glial cells are particularly sensitive to toxic Mn levels, which along with ammonia causes neuroinflammation. Mn activation of NLR family pyrin domain containing 3 (NLRP3) inflammasome is also of particular interest in disease propagation, a multiprotein promoting neuroinflammation. The study done by Sarkar et al. on mouse microglial cells pointed how Mn acts as a signal for activating NLRP3 inflammasome, with subsequent cleavage of caspase 1 and release of inflammatory cytokine interleukin-1β (IL-1β), NLRP 3 activation was linked to mitochondrial dysfunction, O-2 generation, and lysosomal dysfunction [39]. Cell-to-cell propagation of NLRP3 inflammasome and apoptosis-associated speck-like protein (ASC) was noted promoting neuroinflammation, these exosomes can even cross the BBB [39]. This communication as intracellular cargo is similar to the oligomerized transfer of α-Syn and these exosomes can even cross the BBB. Given the high levels of Mn and its access to astrocytic mitochondria, astrocytes are particularly sensitive too, it causes impairment of cellular respiration in astrocytic mitochondria, along with decreased production of glutathione peroxidase. In a literature review done by Sidoryk et al., they emphasized the Mn effect on the glutamine/glutamate, GABA cycle (GGC), it causes impairment of GGC cycle by increasing glutamate extracellularly while decreasing glutamine synthetase activity (GS), depleting glutamine and causing impairment of glutaminergic and gabaergic transmission between astrocytes and neurons in the basal ganglia [40]. Increased glutamate causes excitotoxicity via N-methyl-D-aspartate receptor (NMDA) activation, leading to impaired voluntary movements seen in parkinsonism [40]. High levels of Mn competes with intracellular Ca+2 binding sites in the mitochondria triggering astrogliosis [25]. Mn is also thought to stimulate microglia, releasing inflammatory cytokines and causing upregulation of leucine-rich repeat kinase 2 (LRRK2), which is involved in various neurodegenerative diseases. In a recent study done by Chen et al. on the animal model, leucine-rich repeat kinase 2 inhibitor 1 (LRRK2 IN 1) not only reduced inflammatory cytokines but also recovered the apoptotic function of neuroglia [41].

Moreover, these are recent studies done within the last seven years signifying varied mechanisms by which Mn causes neuroinflammation, its effects on astrocytes damage astrocytic mitochondria which leads to impaired cellular oxidation, and neurotransmitter dysfunction causes impaired cell signaling. The recent study of Sarkar et al. on mouse glial cells is promising in providing an insight into how NLRP3 inflammasome propagates neuroinflammation and neurodegeneration [39]. The role of LRRK2 in association with Mn was a recent study showing its involvement in neuroinflammation via neuroinflammatory cytokines; both of these were animal studies. While the literature review by Sidok et al. determined high Mn levels causing derangements of neurotransmitters [40]. 

6*. Mn and Efflux Proteins*

There are four proteins identified for the transport of Mn out of the cell: ferroportin (Fpn), Solute Carrier Family 30 Member 10 (SLC30A10) located in the cell membrane, secretory pathway Ca ATPase 1 (SPCA1) located in Golgi, Ca/Mn ion pump, ATP13A2 or PARK 9 (ATPase 13A2) located in lysosomes, indirectly regulating Mn transporting Mn from the cytosol to lysosomal membrane [25].

Recently, efflux proteins have gained importance. They play a pivotal role in maintaining Mn levels, preventing neurodegeneration. Of the many proteins involved in the efflux of Mn from the cell, Fpn and SLC30A10 are studied more recently. Fpn mRNA was found to be upregulated in mice upon exposure to Mn as evidenced in a study done by Troadec et al. and there was reduced Mn in the cerebellum of mice [42,43]. SLC30A10 is another protein located on the cell surface involved in Mn efflux. It is primarily located in CNS, gastrointestinal tract (GIT), and liver. In a recent study done by Taylor et al., they signified the importance of this protein in regards to its location, as Mn is primarily excreted in the bile, SLC30A10 is also located in the GIT, liver, and brain (basal ganglia and neurons) making it a crucial regulator in detoxifying Mn [44]. They used two mice phenotypes, one with whole-body Slc30a10 knock out (KO) and the other with tissue-specific KO and the results were astounding showing how the liver and the GIT, including the esophagus, intestines intricately regulates Mn in the brain, concluding that under basal conditions it is the entire GIT regulating Mn and not just the brain or the liver. Only mice with whole-body KO manifested significant alterations in neurobehaviour, then the endoderm specific KO. Next, they also compared the pan/neuronal KO mice with the littermate control and exposed them to high levels of Mn for four weeks, results showed significantly high levels of Mn in pan-neuronal/glial KO, signifying that when there is high Mn concentration, brain SLC30A10 is involved in protecting against neurotoxicity [44]. Recent studies have linked a rare genetic disorder linked to mutation of the SLC30A10 gene, where the loss of function mutation of SLC30A10 results in a syndrome of hepatic cirrhosis, hypermagnesemia, dystonia, polycythemia resulting in familial parkinsonism [20]. These recent studies demonstrated the impact on CNS in the absence of this transporter.

The initial two studies in regards to the effect of Mn on Fpn were done in HEK cells making it more reliable since it was done in a human cell line showing how Fpn, an iron exporter exports Mn, whereas the study done by Taylor et al. is a more recent aggressive study where a cohort study on rats was conducted signifying how Mn and SLC30A10 interact at different Mn concentration [44]. Mechanistic regulation of GIT SLC30A10 under basal condition was a recent discovery. 

Management, diagnosis, and treatment

Conventional Approach

Since the diagnosis of Parkinsonism is difficult in advanced liver cirrhosis, researchers have identified the importance of MRI in patients with chronic liver disease who present with neurocognitive deterioration. Bilateral T1 hyperintensity in globus pallidus is a unique finding in manganism owing to the high concentrations of Mn deposition in these areas. In a case report by Criswell et al. reduced striatal uptake of F DOPA, equally reduced in both caudate and putamen was observed in a patient with advanced liver cirrhosis, signifying characteristic involvement which is different from what is observed in occupational Mn exposure and idiopathic Parkinson’s disease [16]. An observational study by Maffeo et al. signified the importance of MRI in coronary atherosclerotic heart disease (CAHD), with around 28% of patients having T1 hyperintensity on MRI (26/90), representing an essential element for clinical features. They measured Mn blood levels but didn’t find any correlation between blood Mn levels and clinical features [14]. Similarly, a database review by Shin et al. demonstrated pallidal T1 hyperintensity in all the patients with AHD [3].

This characteristic finding leads to the discovery of parkinsonism in cirrhosis and helps in the early identification which will help physicians start early intervention and prevent it from progressing. The reduced uptake by positron emission tomography (PET) imaging is a means to identify altered dopaminergic neurotransmission in patients presenting with advanced liver disease with characteristic localization of globus pallidus and striatum. An observational study also seems powerful in identifying the importance of performing MRI.

Treatment of this debilitating disease is challenging, and currently no treatment has gained promise. Levodopa has shown varied results in AHD and has not shown to be as good as it is in idiopathic Parkinson's disease. In a recent double-blind controlled trial by Sahney et al., bromocriptine for 12 weeks was shown to be effective in mild to moderate symptoms of parkinsonism, with improvements in rigidity and bradykinesia, it was compared with a placebo [45]. Different modalities of treatment can be thought of according to Mn’s effects on the cellular level as shown in Figure 2. In a study on human neuroblastoma cell line SHS-5Y glutathione (GSH) and N acetylcysteine (NAC) were shown to be neuroprotective as an antioxidant [46].

Given the importance of SLC30A10, efflux protein, drugs that enhance the activity of SLC30A10 will help in nonfamilial forms of Mn induced-parkinsonism. Thus efflux of Mn by this transporter would seem to be very effective however more research needs to be done if such therapy will prove beneficial [20]. Similarly, synthetic compounds like ibuprofen, 5 aminosalicylic acids, and para-aminosalicylic acid were shown to protect against mitochondrial damage and apoptosis in vivo and invitro [47,48]. Recently raloxifene, a selective estrogen receptor modulator (SERM) enhanced the expression of Glutamate Aspartate Transporter (GLAST) and glutamate transporter (GLT 1) which take up most of the glutamate from the synaptic cleft to prevent excitotoxic neuronal death. Raloxifene works at the transcriptional level by increasing the expression of GLT1 mRNA and GLAST protein levels. This study was done on rat primary astrocytes by Karki et al. [49]. Valproic acid, an anti-epileptic which modulates GABA neurotransmission has also gained promise in a recent study done by Johnson et al. on mice where valproic acid was found to be neuroprotective, by a variety of mechanisms, it increased expression of glutamate transporters (GLAST and GLT-1) by increasing mRNA and protein levels of these transporters, it also acts as histone deacetylase inhibitor in astrocytes and mouse brain tissue and also reversed Mn induced decrease in TH rendering the protection of the cells against glutamate-induced excitotoxicity, and protecting against dopaminergic neurodegeneration [50].

**Figure 3:**
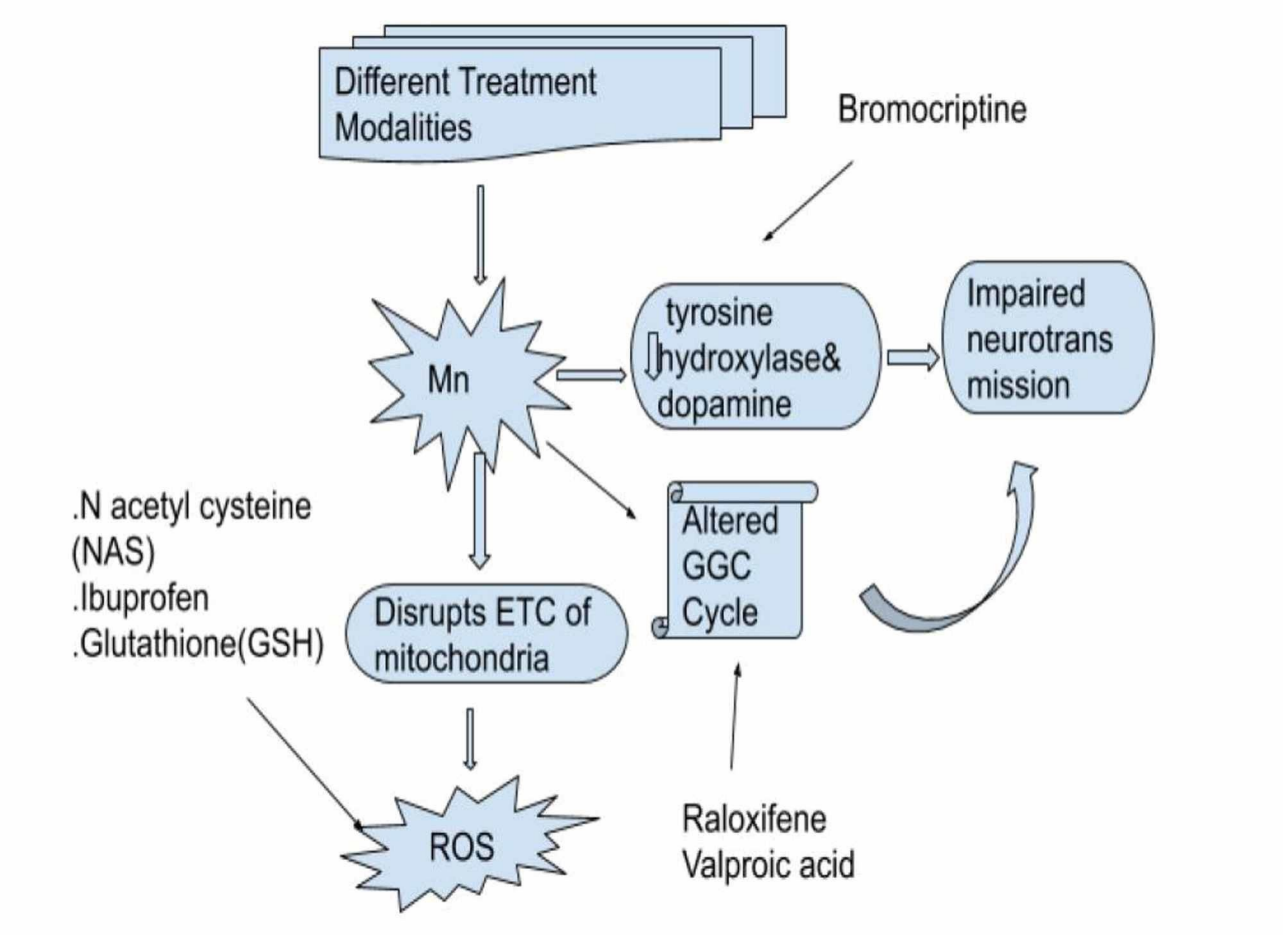
Treatment options for acquired hepatocerebral degeneration. Mn: Manganese; ROS: reactive oxygen species; GGC: glutamine/glutamate GABA cycle

All of these studies are working on simply protecting Mn-induced neurodegeneration outlining the importance of cellular effects of Mn and then working on inhibiting its action by promoting drugs that alter Mn mechanisms, bromocriptine is an inexpensive drug that when used in the milder symptoms has shown good results, the study was done in humans recently and was randomized control trial (RCT) making the study powerful. Similarly, the use of GSH, N acetylcysteine, and ibuprofen working as an antioxidant and a radical scavenger is older comparative studies done on immortalized rat brain cells but it can be used for managing this debilitating illness simply and more feasibly since they are readily available making it a reliable study. The use of raloxifene and valproic acid which modulates glutaminergic neurotransmission are other revolutionary drugs in maintaining homeostatic levels of neurotransmitters thus protecting against neurodegeneration. These studies were also done on rat brain. The effects of the drugs are depicted in Figure 3.

Interventional Approach

If symptoms progress or the clinical diagnosis is delayed, a liver transplant is the last resort with preferably good results in different studies done recently. Orthotopic donor liver transplant has been shown to reverse neurological signs and MRI findings much better than medical treatment alone. An observational study by Maffeo et al. demonstrated reversal of clinical (tremor, dyskinesia, ataxia) and radiological features with the absence of MRI signal at 24 months of follow up in 61% of patients who underwent liver transplantation [14]. Similarly, case reports by Pigoni et al. and Stracciari et al. demonstrated complete symptom recovery with somewhat absence of pallidal hyperintensity at six months and 12 months of follow up [10,17].

These results signify the benefits of a liver transplant, which should be undertaken depending upon the clinical scenario. A study by Mafeo et al., being observational seems to be a powerful recent study [14]. Table 2 lists the important studies that were referenced in this article.

**Table 2:** Table of studies relevant to Mn pathophysiology and management. Mn: manganese; DAT: dopamine transporter; HEK: human embryonic kidney; α-Syn: alpha-synuclein; SPECT: single-photon emission tomography; MRI: magnetic resonance imaging; D2: Dopamine 2; CNS: central nervous system

Author	Year of Publication	Study design /Type of study	Journal	Topic	Findings
Roth J.A et al [12]	2013	Control and DAT transfected HEK cells	Neurotoxicology	Effect of Mn on dopamine toxicity and DAT in control and DAT transfected cells	Dopamine was found to be toxic to the cells with the additive effect with Mn. DAT internalization was also observed
Harischchandra D.S et al [13]	2015	N27 dopaminergic neuronal cell model	Toxicology Sciences	α-Syn protects against Mn neurotoxic insult during the early stages of exposure	α-Syn was neuroprotective during the initial period of exposure
Chen P et al [20]	2015	Review	Journal of Neurochemistry	Mn homeostasis in the nervous system	Mn levels are regulated by specific transporters including importers and exporters.
Shin HW et al [3]	2017	Review	Tremor and other Hyperkinetic movements NY	Recent updates on acquired hepatocerebral degeneration	Multiple mechanisms are involved in acquired hepatocerebral degeneration with Mn, ammonia, and neuroinflammation coming in to play along with T1 hyperintensity on MRI which is a diagnostic feature.
Tryc AB et al [7]	2013	Observational study	Journal of Hepatology	Cirrhosis related Parkinsonism, prevalence, mechanisms and response to treatment	Results showed cirrhosis related parkinsonism in nine of 214 patients (4.2%), SPECT showed decreased DAT and decreased D2 receptor availability. Temporary response to levodopa was 50%, 50% did not even respond to a liver transplant.
Harischandra DS et al [26]	2019	Review	Frontiers in Neuroscience	Manganese induced neurotoxicity, new insights into the triad of protein misfolding, mitochondrial impairment and neuroinflammation	Manganese effects on CNS, Mn was found to impair mitochondrial integrity, oligomerization of α-Syn and promotes neuroinflammation

Limitations

In patients who cannot undergo a liver transplant. Our synthesis of literature indicates the association of manganese with advanced liver disease, sharing common clinical features of Parkinson’s disease, yet pathologically different. Number of the published articles on the direct assessment of cirrhotic patients presenting with Parkinson’s disease with Mn pathophysiology was limited. Most of the literature retrieved to understand the Mn effect was from the articles signifying Manganism which occurs after environmental or occupational exposure of manganese. Most are animal studies done on rats, except for a few clinical trials and case reports, where patients presented with extrapyramidal symptoms lead to the management of this debilitating disease. More observational studies need to be conducted on a larger population to identify the progress of liver transplantation in reversing clinical and radiological features. Larger observational studies should be conducted on humans, as this debilitating disease needs more attention so that it can be prevented in the first place. More clinical trials should be done to effectively treat this disease.

## Conclusions

Acquired hepatocerebral degeneration, with the involvement of Mn in its pathogenesis, is a severely debilitating disease, seen in advanced liver disease. Regulation of Mn is tightly regulated but with advanced liver disease and portosystemic shunting, renders the body to toxic accumulation of chemicals and neurotoxins. With Mn being one of them we delineated how Mn from its influx has varied effects on major organelles in the cytoplasm, effects membrane transporters (DAT), and neurotransmitters mediating neurotoxicity and neuroinflammation. We also touched on the management of this disease, which is complex with varied results. Since the prognosis of liver cirrhosis is generally poor, the neurological complication that intervenes may require targeted treatment. Given the molecular mechanisms we now know of Mn, we have set grounds for future scientists to work on identifying drugs that can either work on preventing its influx, mediate its efflux by working on SLC30A10 expression, drugs that can inhibit α-Syn oligomerization and propagation, where exosomes can act as drug-delivering machines, antimetabolites which can protect against oxidative stress or drugs that can mediate neurotransmitters. Larger clinical trials will be beneficial for our community and will help prevent patients from undergoing liver transplants especially for patients with advanced age and comorbidities who cannot handle this interventional approach. More cohort studies need to be conducted on a larger scale where patients with liver cirrhosis should be followed to identify both clinical and radiological changes early on to prevent its progression so that it can be treated with the best treatment strategy based on individual needs.
